# The widespread action observation/execution matching system for facial expression processing

**DOI:** 10.1002/hbm.26262

**Published:** 2023-03-09

**Authors:** Wataru Sato, Takanori Kochiyama, Sakiko Yoshikawa

**Affiliations:** ^1^ Psychological Process Research Team Guardian Robot Project, RIKEN Kyoto Japan; ^2^ Brain Activity Imaging Center, ATR‐Promotions Kyoto Japan; ^3^ Kyoto University of the Arts Kyoto Japan

**Keywords:** amygdala, cerebellum, dynamic facial expressions of emotion, facial nerve nucleus, group independent component analysis (ICA), mirror neuron system

## Abstract

Observing and understanding others' emotional facial expressions, possibly through motor synchronization, plays a primary role in face‐to‐face communication. To understand the underlying neural mechanisms, previous functional magnetic resonance imaging (fMRI) studies investigated brain regions that are involved in both the observation/execution of emotional facial expressions and found that the neocortical motor regions constituting the action observation/execution matching system or mirror neuron system were active. However, it remains unclear (1) whether other brain regions in the limbic, cerebellum, and brainstem regions could be also involved in the observation/execution matching system for processing facial expressions, and (2) if so, whether these regions could constitute a functional network. To investigate these issues, we performed fMRI while participants observed dynamic facial expressions of anger and happiness and while they executed facial muscle activity associated with angry and happy facial expressions. Conjunction analyses revealed that, in addition to neocortical regions (i.e., the right ventral premotor cortex and right supplementary motor area), bilateral amygdala, right basal ganglia, bilateral cerebellum, and right facial nerve nucleus were activated during both the observation/execution tasks. Group independent component analysis revealed that a functional network component involving the aforementioned regions were activated during both observation/execution tasks. The data suggest that the motor synchronization of emotional facial expressions involves a widespread observation/execution matching network encompassing the neocortex, limbic system, basal ganglia, cerebellum, and brainstem.

## Graphical abstract

Conjunction analyses revealed that, in addition to neocortical regions (i.e., the right ventral premotor cortex and right supplementary motor area), bilateral amygdala, right basal ganglia, bilateral cerebellum, and right facial nerve nucleus were activated during both the observation/execution of facial expression tasks. Group independent component analysis revealed that a functional network component involving the aforementioned regions was activated during both the observation/execution tasks. The data suggest that the motor synchronization of emotional facial expressions involves a widespread observation/execution matching network encompassing the neocortex, limbic system, basal ganglia, cerebellum, and brainstem.

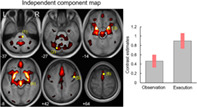

## 1 INTRODUCTION

Observing and understanding others' emotional facial expressions plays a primary role in face‐to‐face communication (Mehrabian, 1971). There has been a great deal of interest among scholars, including philosophers (e.g., Nietzsche, 1997) and psychologists (e.g., Lipps, 1903), in the psychological mechanisms of face‐to‐face communication, and it has been speculated that the motor synchronization involved in facial expressions or facial mimicry is important for understanding others' emotional states. Consistent with this idea, previous behavioral studies showed that the observation of others' facial expressions automatically elicits congruent facial muscular activity in the observer (e.g., Dimberg, 1982). Such facial muscle activity is related to the elicitation and recognition of emotional states (Sato et al., 2013). Studies also showed that motor synchronization facilitates social bonding by promoting liking and rapport (Kulesza et al., 2015).

To investigate the neural correlates of the motor synchronization of facial expressions, a few previous functional magnetic resonance imaging (fMRI) studies have investigated brain regions active during both the observation/execution of emotional facial expressions (Anders et al., 2012; Hennenlotter et al., 2005; Kircher et al., 2013; Krautheim et al., 2020). Such studies were based on single‐unit recording studies, which revealed neurons in the ventral premotor cortex (PMv) that discharge when a monkey performs specific actions, and when it observes experimenters performing similar actions; these neurons were named mirror neurons (Grafton et al., 1996; Rizzolatti et al., 1996; for reviews, see Bonini et al., 2022; Heyes & Catmur, 2022). Subsequent neuroimaging studies in humans confirmed that some brain regions, including the PMv, are active during both the observation/execution of hand or foot movements (Cerri et al., 2015; Chong et al., 2008; Cunnington et al., 2006; Errante & Fogassi, 2020; Errante et al., 2021; Gazzola & Keysers, 2009; Grèzes et al., 2003; Hétu et al., 2011; Hotz‐Boendermaker et al., 2011; Kilner et al., 2009; Molenberghs et al., 2010; Simos et al., 2017; Thioux & Keysers, 2015; Turella et al., 2009; for reviews, see Hardwick et al., 2018; Molenberghs et al., 2012); these regions were proposed to constitute an action observation/execution matching system or mirror system (Gallese et al., 1996; Rizzolatti et al., 1996, 2001). Based on these data, three studies investigated brain activation common to both the observation of dynamic emotional facial expressions versus neutral expressions, and the execution of facial expressions versus rest (Anders et al., 2012; Hennenlotter et al., 2005; Kircher et al., 2013; Krautheim et al., 2020). These studies demonstrated consistent activation of the right PMv, including the precentral gyrus and inferior frontal gyrus, by conducting conjunction analysis (Anders et al., 2012; Kircher et al., 2013; Krautheim et al., 2020) or evaluating the spatial overlap in activity (Hennenlotter et al., 2005). These data suggest that the PMv is involved in the matching between observation/execution of actions associated with emotional facial expressions, and constitutes the action observation/execution matching system for facial expression processing.

However, it remains unclear whether other brain regions could be involved in such a matching system for processing facial expressions; results have been inconsistent and some brain regions were not explored. First, we expected the amygdala to be involved in this process, because two studies (Kircher et al., 2013; Krautheim et al., 2020) reported activation thereof in the context of both the observation/execution of facial expressions, although other studies (Anders et al., 2012; Hennenlotter et al., 2005) did not observe this. Several previous fMRI studies reported amygdala activation during the observation of facial expressions (for a review, see Liu et al., 2021), and some studies reported that its activation was associated with the production of emotional facial expressions (Heller et al., 2014). Second, the cerebellum could be relevant; its activation thereof was reported in both observation/execution tasks in two studies (Kircher et al., 2013; Krautheim et al., 2020), although this was not seen in other studies (Anders et al., 2012; Hennenlotter et al., 2005). Several previous fMRI studies reported that this region is active during the observation of dynamic facial expressions of emotion (Sato et al., 2019). One fMRI study that measured facial electromyography in response to dynamic facial expressions reported that cerebellar activity was associated with facial mimicry (Likowski et al., 2012). Some previous studies that assessed shared neural activity between the observation/execution of hand movements reported activity in the cerebellum (Errante et al., 2021; Errante & Fogassi, 2020; Gazzola & Keysers, 2009). Finally, the brainstem could be part of the action observation/execution matching system for facial expression processing, because the facial nerve nucleus (facial motor nucleus) in the brainstem is critical for the execution of facial movements. This region, from which the facial nerve (the seventh cranial nerve) projects, is the final part of the brain circuit underlying facial muscle control (for a review, see Cattaneo & Pavesi, 2014). A previous fMRI study showed that deliberate facial muscle activity activated the facial nerve nucleus (Komisaruk et al., 2002). The involvement of these regions outside of the classic action observation/execution matching system could be in line with the theoretical proposal that not only motor; however, other types of information (e.g., emotional) may also be processed through an embodied simulation (Gallese, 2005; Gallese et al., 2004; Gallese & Caruana, 2016; Rizzolatti et al., 2014), and that motor simulation could modulate emotion‐related brain activity (Wood et al., 2016). In summary, we hypothesized that activity common to the observation/execution of facial expressions would be found not only in the neocortical observation/execution matching system in the right PMv, but also in the amygdala, cerebellum, and facial nerve nucleus.

Furthermore, whether the widespread action observation/execution matching regions constitute the functional network underlying the observation/execution of facial expressions remains to be tested. To the best of our knowledge, no previous study has tested the functional or effective connectivity patterns commonly associated with the observation/execution of emotional facial expressions. Although some studies have investigated the action observation/execution matching network involved in the processing of facial expressions using related tasks, such as observation (Arioli et al., 2018; Sato et al., 2012, 2017; Voon et al., 2010), emotional attribution (Spunt & Lieberman, 2012), and imitation (Sadeghi et al., 2022) tasks, which have revealed inter‐regional connectivity, the target brain areas have been restricted to certain observation/execution matching regions, such as the PMv and amygdala. Although a few studies investigated the involvement of the shared network in the observation/execution of hand gestures (Sasaki et al., 2012; Simos et al., 2017) and reported evidence of coupling among action observation/execution matching regions, those studies only evaluated neocortical regions (e.g., PMv). Anatomical studies in animals demonstrated connections among brain regions, including the PMv–amygdala (Avendano et al., 1983), PMv–cerebellum (Dum & Strick, 2003), PMv–facial nerve nucleus (Morecraft et al., 2001), amygdala–cerebellum (Çavdar et al., 2022), amygdala–facial nerve nucleus (Van Daele et al., 2011), and cerebellum–facial nerve nucleus (Kotchabhakdi & Walberg, 1977). Based on these data, we hypothesized that widespread action observation/execution matching regions, including the PMv, amygdala, cerebellum, and facial nerve nucleus, could constitute a functional network involved in both the observation/execution of facial expressions.

To investigate these hypotheses, we acquired fMRI data during the observation of dynamic facial expressions and execution of facial actions associated with emotional facial expressions. During observation tasks, we presented participants with videos of facial expressions of anger and happiness (negative and positive emotional valence, respectively), as well as dynamic randomized mosaic images. During execution tasks, we presented plus signs in various colors and asked participants to activate the corrugator supercilii (brow lowering; typically seen in angry facial expressions) and zygomatic major muscles (lip corner pulling; typically seen in happy facial expressions) and to rest. We performed conjunction analysis to identify commonalities in regional brain activity during the facial expression observation/execution tasks. To investigate the functional network, we conducted group independent component analysis (ICA) of fMRI data to analyze functional connectivity (Calhoun et al., 2001). Functional connectivity refers to the temporally coordinated activity of spatially distant regions (Engel, 2021). ICA is one of the two major methods for evaluating functional connectivity; the other is seed‐based correlation (Joel et al., 2011). The seed‐based method is hypothesis‐driven and efficient for determining the restricted connectivity of a selected seed region to the rest of the brain, whereas ICA is data‐driven and efficient for examining general connectivity patterns across the whole brain (Wu et al., 2018). We expected to find the widespread functional network component, which could include the abovementioned regions and be activated during both observation/execution tasks.

## 2 METHODS

### 2.1 Participants

The study included 23 volunteers (10 women; mean ± SD age, 29.9 ± 6.4 years). We estimated the sample size via a priori power analysis using NeuroPower (Durnez et al., 2016). We randomly selected data (*n* = 20) from our previous study using an observation task (dynamic facial expressions vs. dynamic mosaics) (Sato et al., 2019). We assumed a one‐sample *t* test, with an *α* level of 0.05 and power of 0.80 based on random field theory. The results showed that 21 participants were required. We also heuristically (Lakens, 2022) referred to some previous fMRI studies that reported action observation/execution matching regional activity during both the observation/execution of face or hand movements using conjunction analysis with a global null hypothesis (*n* = 16–18; Anders et al., 2012; Errante & Fogassi, 2020; Errante et al., 2021). All participants were right‐handed, as revealed by the Edinburgh Handedness Inventory (Oldfield, 1971), and had normal or corrected‐to‐normal visual acuity. After the experimental procedures had been fully explained, all participants provided written informed consent, which was approved by the Ethics Committee of Brain Activity Imaging Center, ATR‐Promotions. This study was conducted in accordance with the guidelines provided by the Brain Activity Imaging Center, ATR‐Promotions. Experiments were conducted in 2016.

### 2.2 Experimental design

A within‐subject design was used. The observation task included two factors: stimulus condition (expression or mosaic) and emotion (anger or happiness). The execution task included one factor: action condition (corrugator supercilii action, zygomatic major action, or rest).

### 2.3 Stimuli

**FIGURE 1:**
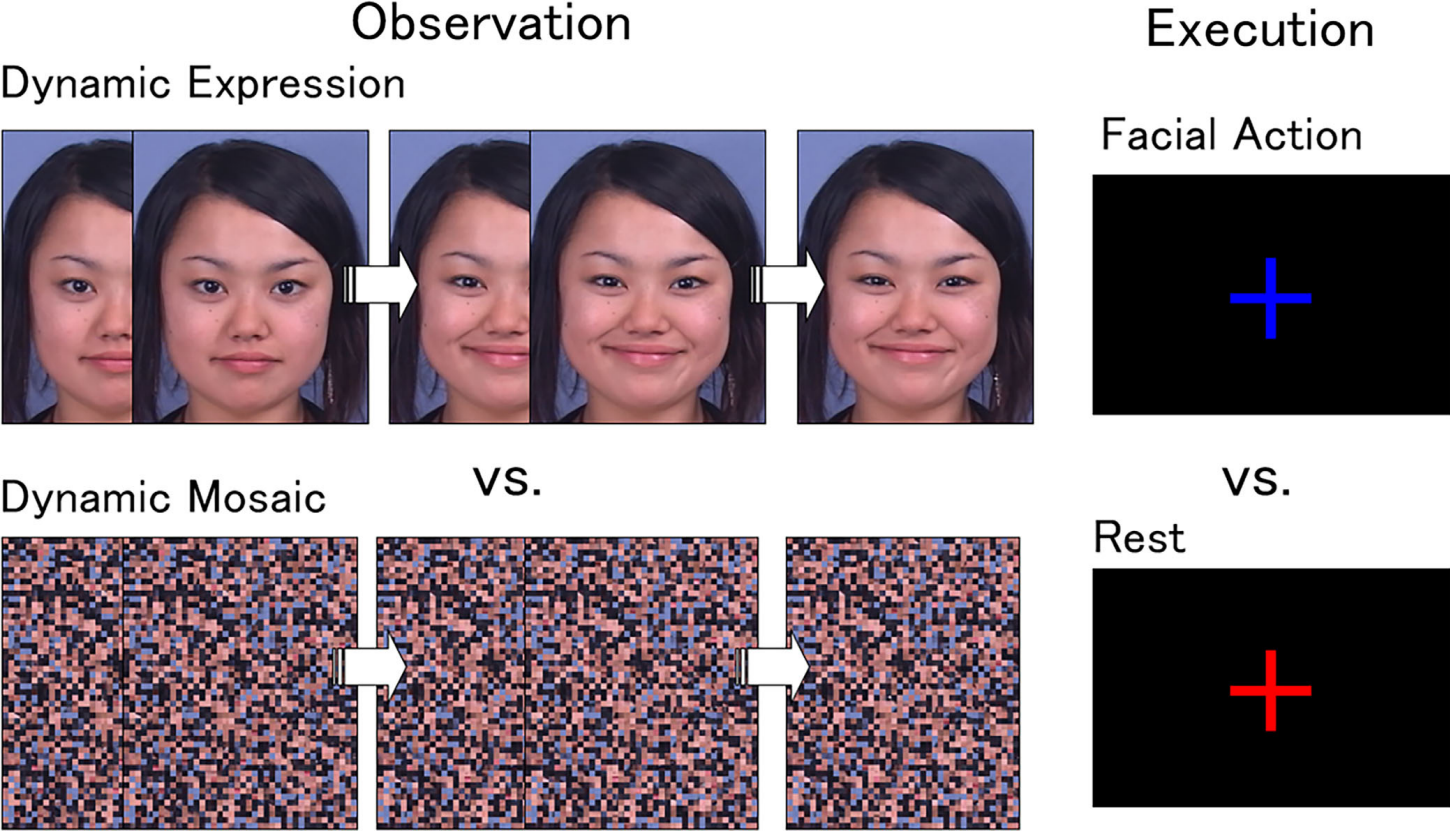
Illustrations of the facial expression observation/execution tasks. During the observation task, participants passively observed angry or happy dynamic facial expressions, or dynamic random mosaics. During the execution task, plus symbols of three different colors were presented, and participants activated specific facial muscles (corrugator supercilii or zygomatic major) or rested.

The dynamic facial expression stimuli in the observation task, consisted of videos of four women and four men making angry and happy facial expressions (Figure 1). These stimuli were selected from our video database of facial expressions of emotion that includes 65 Japanese models. The stimulus model looked straight ahead. All of the faces were unfamiliar to the participants. The stimuli were selected because they were considered representative of the facial expressions of interest, as confirmed by analyses performed by a trained coder using the Facial Action Coding System (FACS) (Ekman & Friesen, 1978) and FACS Affect Interpretation Dictionary (Ekman et al., 1998). In addition, the speed of the dynamic changes in these expressions was within the natural range for observers (Sato & Yoshikawa, 2004). The stimuli have been validated in several previous behavioral studies, i.e., were identified as angry or happy expressions (Sato et al., 2010), and elicited appropriate subjective emotional responses (Sato & Yoshikawa, 2007b) and spontaneous facial mimicry (Sato et al., 2008; Sato & Yoshikawa, 2007a; Yoshimura et al., 2015). The dynamic expression stimuli comprised 38 frames ranging from neutral to emotional expressions. Each frame was presented for 40 ms and each clip for 1520 ms. The stimuli subtended a visual angle of ~15° vertical × 12° horizontal. An example stimulus sequence is shown in Figure 1, which includes data from a model who provided consent to the use of her image in scientific publications.

For the dynamic randomized mosaic image stimuli, all of the dynamic facial expression frames were divided into 50 vertical × 40 horizontal squares and reordered using a fixed randomization algorithm (Figure 1). This rearrangement rendered each image unrecognizable as a face. A set of 38 of these frames corresponding to the original dynamic face images was serially presented (without changing the frame order) as a moving clip at the same speed as the dynamic expression stimuli. The resultant unrecognizable dynamic mosaic stimuli were presented with smooth motion comparable to natural dynamic facial expressions.

For the execution task, red, blue, and green plus symbols that subtended a visual angle of ~3° vertical × 3° horizontal were used.

### 2.4 Presentation apparatus

The experimental events were controlled by Presentation Software (version 10.0; Neurobehavioral Systems), implemented on a computer running Microsoft Windows (Microsoft Corp.). The stimuli were projected from a liquid crystal projector (DLA‐G150CL; Victor Electronics) to a mirror positioned in a scanner in front of the participant, at a refresh rate of 75 Hz.

### 2.5 Procedure

We tested the action observation/execution matching system or mirror neuron system for facial expressions using observation/execution tasks, based on the original definition that the system should be activated during both the observation/execution of actions (Rizzolatti et al., 2001). Although some previous studies have used the imitation task to investigate this issue (e.g., Sadeghi et al., 2022), we did not include it, mainly to simplify the experimental design but also because that task may not clearly indicate whether activation during action execution is driven by visual input or executed action (Kircher et al., 2013).

The tasks were tested in two separate fMRI runs because each task required different responses and instructions. The total time for each run was 747 s, consisting of 20 on‐epochs for 24 s, during which experimental stimuli were presented, interspersed with 21 off‐epochs for 12 s, during which a fixation point was presented in the center of the screen. The initial 15 s were discarded, as the signal was reaching a steady state. The order of the observation/execution task (one run each) was counterbalanced across participants. Each experimental condition (observation: angry‐expression, happy‐expression, angry‐mosaic, and happy‐mosaic; execution: corrugator‐supercilii‐action, zygomatic major‐action, and rest) was presented during each on‐epoch, within each run. The order of epochs within each run was counterbalanced across participants. Before fMRI image acquisition, participants completed several practice trials for familiarity with the activation of specific facial muscles, using a procedure similar to one described previously (Hyniewska & Sato, 2015). The participants observed figures showing the anatomical locations of the corrugator supercilii and zygomatic major muscles, along with figures and videos activating these muscles. Then, they were asked to activate the facial muscles themselves by an experimenter trained in the FACS. The experimenter observed and corrected any errors in participants' facial muscle activity. No emotional or related terms, such as “smile” or “frown,” were used to describe the facial muscle activity.

During each trial in the observation task, after presentation of a fixation point (a small white plus symbol that subtended a visual angle of ~1° vertical × 1° horizontal in the center of the black screen) for 1500 ms, the dynamic facial expression or dynamic random mosaic stimulus was presented for 1500 ms. Participants were instructed to fixate on the fixation point and passively observe the stimuli.

During each trial in the execution task, after the presentation of a fixation point that was the same as in the observation task, a large plus symbol in one of three colors was presented for 1500 ms. Participants were instructed to fixate on the fixation point and activate (and maintain activation) specific facial muscles (corrugator supercilii or zygomatic major), or to rest, during presentation of the colored plus symbol. The association between colors and conditions was explained before the tasks and counterbalanced across participants.

To ensure that the participants could appropriately activate the facial muscles in response to the instructions, we repeated the execution task after MRI data acquisition (with video recording). The participants completed a total of four on‐epochs for 24 s, during which experimental stimuli were presented as in the fMRI scan, interleaved with three off‐epochs of 15 s. Evaluation by a scorer trained in FACS coding confirmed that all participants appropriately activated the corrugator supercilii or zygomatic major, or rested, according to the instructions.

### 2.6 MRI acquisition

Imaging was performed using a 3‐T scanning system (MAGNETOM Prisma, Siemens Medical Solutions) at the ATR Brain Activity Imaging Center using a 64‐channel head coil. Small elastic pads were placed on both sides of the head to stabilize its position. The functional images consisted of 76 consecutive axial slices parallel to the anterior commissure–posterior commissure plane, without a gap and covering the whole brain, including the brainstem. A T2*‐weighted multiband gradient‐echo echo‐planar imaging (EPI) sequence was used with the following parameters: repetition time (TR) = 1500; echo time (TE) = 30 ms; flip angle = 60°; multiband acceleration factor = 4; partial Fourier = 6/8; matrix size = 96 × 96; voxel size = 2 × 2 × 2 mm. At the beginning of each fMRI run, we acquired a gradient‐echo field map to correct for geometric distortions (TR = 738 ms; TE1/TE2 = 4.92/7.38 ms [ΔTE = 2.46 ms]; flip angle = 60°; matrix size = 96 × 96; 76 slices in the same orientation and geometry as the EPI sequence). After acquiring the functional images, a T1‐weighted high‐resolution anatomical image was obtained using a magnetization‐prepared rapid‐acquisition gradient‐echo (MPRAGE) sequence (TR = 2250 ms; TE = 3.06 ms; flip angle = 9°; field of view = 256 × 256 mm; voxel size = 1 × 1 × 1 mm).

### 2.7 Image analysis

Image analyses were performed using the Statistical Parametric Mapping package (SPM12; revision 7487; http://www.fil.ion.ucl.ac.uk/spm), implemented in MATLAB R2018 (MathWorks).

For preprocessing, all functional images were first corrected for slice timing. Next, functional images of each run were realigned using the first scan as a reference to correct for head motion, and were unwarped to correct for distortions based on the field map, and movement and distortion occurring during image acquisition, using FieldMap Toolbox (Andersson et al., 2001; Hutton et al., 2002). The realignment parameters revealed only a small amount of motion correction (<1.5 mm for the observation task and <2 mm for the execution task). Then, the functional images were coregistered to the skull‐stripped anatomical image. Subsequently, all anatomical and functional images were spatially normalized to Montreal Neurological Institute (MNI) space using the anatomical image‐based unified segmentation‐spatial normalization approach (Ashburner & Friston, 2005). The cerebellum and brainstem normalization were performed using the spatially unbiased infratentorial template (SUIT) toolbox (version 3.4; https://www.diedrichsenlab.org/imaging/suit.htm; Diedrichsen, 2006; Diedrichsen et al., 2009), which provides high‐resolution cerebellum and brainstem‐specific templates and allows for more accurate intersubject cerebellum normalization than the whole‐brain SPM approach. Finally, the spatially normalized functional images were resampled to a voxel size of 2 **×** 2 **×** 2 mm and smoothed with an 8 mm full width at half‐maximum (FWHM) isotropic Gaussian kernel to compensate for anatomical variability among participants. A previous methodological study has shown that the optimal FWHM for general linear model (GLM) group inference according to various criteria (e.g., sensitivity, inter‐participant variability) is 8 mm (Mikl et al., 2008). Another study showed that changes in FWHM values, including 0, 4, and 8 mm, have little effect on the results of group ICA analyses (Alahmadi, 2021).

Random‐effects analyses were performed to identify significantly activated voxels at the population level (Holmes & Friston, 1998). First, we performed a single‐subject analysis using the GLM (Friston et al., 1995). The presentation conditions were embedded within a series of boxcar functions. The task‐related regressor was modeled by convolution with a canonical hemodynamic response function. We used a high‐pass filter composed of a discrete cosine basis function with a cutoff period of 128 s to eliminate the artifactual low‐frequency trend. To reduce motion‐related noise and other noise confounders, such as physiological noise (e.g., respiratory, cardiac, or vascular activity), additional nuisance regression was conducted using the PhysIO Toolbox (version 3.2.0; Kasper et al., 2017), which is part of the Translational Algorithms for Psychiatry‐Advancing Science (TAPAS) software collection (https://translationalneuromodeling.github.io/). The nuisance regressors included six head motion parameters generated by the realignment step and six cerebrospinal fluid (CSF)‐related time courses. CSF signals across voxels in the associated mask were extracted for each participant, and the average and first five principal components of CSF signals were used as nuisance regressors based on the CompCor method (Behzadi et al., 2007). Unlike the standard CompCor method, the white matter (WM) signals were not included to avoid undesirable removal of brain signals from the brainstem nuclei distributed within the WM region in the brainstem. Serial autocorrelation, assuming a first‐order autoregressive model, was estimated from the pooled active voxels with a restricted maximum likelihood procedure, and used to whiten the data and design matrix (Friston et al., 2002). The contrast images of each task condition from the first‐level single‐subject analysis were entered into a full factorial model for the second‐level random effects analysis.

Initially, the contrast between the task and control conditions was examined for each task (dynamic facial expressions versus dynamic mosaics for the observation task and facial muscle activations versus rest for the execution task). Voxels were identified as significantly activated if they reached the height threshold of *p* < .05 with a family‐wise error (FWE) corrected for multiple comparisons over the whole brain at the peak level. For analysis of the cerebellum and brainstem, we used small volume correction (SVC) (Worsley et al., 1996). The regions were defined using the binarized cerebellar mask based on the probabilistic atlas of cerebellar lobules (Lobules‐SUIT.nii) in the SUIT toolbox, and the binarized brainstem mask based on the Harvard‐Oxford cortical and subcortical structural atlases (HarvardOxford‐sub‐maxprob‐thr50‐2mm.nii) (cf https://fsl.fmrib.ox.ac.uk/fsl/fslwiki/Atlases). These were conducted as exploratory analyses for subsequent statistical tests of commonalities.

Next, to test for commonalities in brain activity between the observation/execution tasks, we performed conjunction analysis using a global null hypothesis (Friston et al., 1999), as used in some previous studies of action observation/execution matching system activity during the observation/execution of bodily movements (e.g., Anders et al., 2012; Errante et al., 2021; Errante & Fogassi, 2020; Hotz‐Boendermaker et al., 2011). This approach is valid for the identification of consistent activity across conditions (Friston et al., 2005; however, see Nichols et al., 2005). For this analysis, we tested whether both the contrasts of the observation of dynamic facial expressions versus dynamic mosaics in the observation task and execution of facial actions versus rest in the execution task were consistently high and jointly significant, using the minimum *T*‐statistic. This is equivalent to inferring the presence of one or more effects (Friston et al., 2005). Therefore, it should be noted that our significant conjunction does not indicate that all of the contrasts were individually significant. Voxels were deemed significant according to the same threshold described in the above analysis.

The brain structures were anatomically labeled and identified according to Brodmann's area using the Automated Anatomical Labelling atlas (Tzourio‐Mazoyer et al., 2002) and Brodmann maps (Brodmann.nii) provided by MRIcron software (https://people.cas.sc.edu/rorden/mricron/index.html), respectively. The probabilistic atlas of cerebellar lobules provided by the SUIT toolbox was used for anatomical localization and labeling of cerebellar activations (Diedrichsen et al., 2009). Brainstem activations (e.g., facial nerve nucleus) were localized and labeled by referring to Duvernoy's (1995) atlas as in a previous study (Faull et al., 2015). Using axial images approximately *z* = −38 of the mean structural images of participants, we delineated the anatomical structures of the facial nerve nucleus and its adjacent structures (e.g., the facial nerve and spinal trigeminal nucleus). The area of activation is superposed on mean normalized structural images of the participants in this study to illustrate the anatomical regions in figures.

Finally, to analyze functional connectivity, we conducted group ICA of the fMRI data of both tasks. Before ICA analysis, the spatially preprocessed data were further processed using nuisance regression including the same nuisance regressors included in the above GLM analysis (i.e., 11 discrete cosine transform basis functions acting as a high pass filter, with a cut‐off period of 128 s, six head motion parameters, and six CSF‐related time courses). Then the cleaned data were entered into the GIFT v4.0b software (Calhoun et al., 2001). Principal component analysis was performed to reduce the dimensionality of the fMRI data set at the subject and group levels. To identify independent components common to both the observation/execution tasks at the group level, we conducted temporal concatenation across two task runs and all participants. The informax algorithm (Bell & Sejnowski, 1995) was applied for the group ICA, and estimated 20 independent components. Data were back‐reconstructed using the default GIFT option with *z*‐scoring to create each participant's time courses and spatial maps. The reconstructed time courses of 20 components of all participants were evaluated using random‐effects analyses as in the aforementioned regional activity analyses, with the exception that the design matrix of the first‐level single‐subject analysis included task‐related regressors only. The GIFT utilities “Temporal Sorting” and “Stats on Beta Weights” were used in this analysis. Contrast for the observation of dynamic facial expressions versus dynamic mosaics in the observation task and the execution of facial actions versus rest in the execution task were evaluated. We report only components that were significant for both contrasts. The threshold for significance was set at *p* < .05 (one‐tailed) with Bonferroni correction applied for multiple testing across 20 independent components (i.e., *p* < .05/20). The spatial overlap between independent components and the results of the above conjunction analysis was evaluated by testing whether the activation foci in the conjunction analysis were included in the independent component maps.

## 3 RESULTS

### 3.1 Regional brain activity for each task

The contrasts between the observation of dynamic facial expressions versus dynamic mosaics in the observation task (Table 1), and between the execution of facial actions versus rest in the execution task (Table 2), were individually tested. In both tasks, significant activity was detected in the right PMv, left supplementary motor area (SMA), and bilateral cerebellum. The activity of the bilateral amygdala was detected for the observation task, and also for the execution task when a more liberal height threshold was used (*p* < .001, uncorrected). The bilateral basal ganglia and right facial nerve nucleus were activated during the execution task, as well as during the observation task with a more liberal height threshold (*p* < .001, uncorrected). In addition, we conducted the overlap analysis to test for a logical AND of the observation/execution tasks. The same threshold was applied to the individual task‐related contrasts (a peak‐level FWE corrected threshold was used except for the facial nerve nucleus, where an uncorrected threshold was used) and then the resulting statistical map from the execution task was masked with that of the observation task. The results showed the spatial overlap of the aforementioned regions (Table 3).

**TABLE 1:** Brain regions that exhibited significant activation in the observation task (dynamic facial expressions vs. dynamic mosaics).

			Coordinates				Cluster	
Side	Region	BA	x	y	z	Z‐value	Size (voxel)	MCC
R	Inferior temporal gyrus	37	46	−48	−22	8.04	3747	a
R	Inferior occipital gyrus	19	46	−76	−10	8.04		a
R	Middle temporal gyrus	37	56	−58	4	8.04		a
L	Inferior occipital gyrus	19	−46	−78	−8	8.04	1128	a
L	Fusiform gyrus	37	−42	−50	−20	8.04		a
R	Hippocampus	‐	22	−6	−16	8.04	1096	a
R	Amygdala	‐	28	0	−20	8.04		a
L	Amygdala	‐	−28	−2	−20	5.97	422	a
R	Precentral gyrus	6	46	6	40	6.95	857	a
R	Inferior frontal gyrus	48	44	22	22	5.76		a
R	Inferior frontal gyrus	45	54	30	14	5.74		a
R	Inferior temporal gyrus	20	40	−2	−44	5.43	8	a
L	Lingual gyrus	27	−12	−34	−2	4.96	10	a
R	Supplementary motor area	6	4	18	62	4.80	6	a
R	Cerebellum crus 1	‐	44	−48	−27	6.61	78	b
L	Cerebellum crus 2	‐	−12	−78	−41	5.55	100	b
L	Cerebellum VI	‐	−14	−74	−27	4.92	29	b
L	Cerebellum VIIb	‐	−28	−68	−53	4.47	13	b
L	Cerebellum crus 1	‐	−42	−66	−27	4.37	4	b
R	Facial nerve nucleus	‐	8	−30	−35	3.13	2	c

*Note*: The coordinates of activation foci in the MNI system are shown. a—Family‐wise error corrected for multiple comparisons over the whole brain (*p* < .05). b—Small volume correction over the cerebellum (*p* < .05). c—Uncorrected *p* < .001.

Abbreviations: BA, Brodmann areas; MCC, multiple comparison correction.

**TABLE 2:** Brain regions that exhibited significant activation in the execution task (facial actions vs. rest).

Side	Region	BA	Coordinates			Z‐value	Cluster	MCC
			x	y	z		Size (voxel)	
L	Supplementary motor area	6	−4	−4	62	8.04	2402	a
R	Precentral gyrus	4	50	−8	38	8.04	13542	a
R	Precentral gyrus	6	42	−10	42	8.04		a
R	Rolandic operculum	48	48	0	8	8.04		a
L	Precentral gyrus	3	−44	−14	34	8.04		a
L	Precentral gyrus	4	−50	−8	38	8.04		a
L	Rolandic operculum	48	−48	2	6	8.04		a
L	Precentral gyrus	6	−60	4	32	8.04		a
R	Pallidum	‐	24	−4	4	8.04		a
L	Putamen	‐	−24	−6	4	8.04		a
L	Insula	48	−34	0	6	8.04		a
R	Insula	48	36	6	4	8.04		a
L	Amygdala	‐	−26	−2	−14	4.02	4	d
R	Amygdala	‐	28	−2	−14	3.94	2	d
L	Cerebellum VI	‐	−12	−66	−19	8.04	3727	b
R	Cerebellum VI	‐	14	−66	−21	8.04		b
R/L	Vermis VIIIa	‐	0	−60	−33	6.10		b
L	Cerebellum VIIIa	‐	−32	−58	−53	8.04	804	b
R	Cerebellum VIIIa	‐	34	−58	−55	7.74	675	b
R	Cerebellum VIIb	‐	16	−70	−51	7.28		b
L	Facial nerve nucleus	‐	−4	−38	−43	7.61	413	c
R	Facial nerve nucleus	‐	6	−38	−43	6.66		c

*Note*: The coordinates of activation foci in the MNI system are shown. a—Family‐wise error corrected for multiple comparisons over the whole brain (*p* < .05). b—Small volume correction over the cerebellum (*p* < .05). c—Small volume correction over the brain stem (*p* < .05). d—Uncorrected *p* < .001.

Abbreviations: BA, Brodmann areas; MCC, multiple comparison correction.

**TABLE 3:** Brain regions that exhibited significant activation in the execution (facial actions vs. rest) task masked with the observation (dynamic facial expressions vs. dynamic mosaics) task.

Side	Region	BA	Coordinates			Z‐value	Cluster	MCC
			x	y	z		Size (voxel)	
R	Precentral gyrus	6	50	4	48	8.04	131	a
R	Pallidum	‐	26	−8	−8	5.96	20	a
R	Amygdala	‐	28	−4	−12	5.38		a
L	Amygdala	‐	−24	−2	−12	5.79	7	a
R	Supplementary motor area	6	4	16	64	4.67	1	a
R	Cerebellum crus 1	‐	42	−58	−27	5.55	10	b
L	Cerebellum VI	‐	−18	−72	−27	5.33	4	b
L	Cerebellum crus 1	‐	−42	−66	−27	6.35	4	b
L	Cerebellum VIIb	‐	−28	−66	−53	6.48	5	b
R	Facial nerve nucleus	‐	4	−36	−37	4.98	6	c

*Note*: The coordinates of activation foci in the MNI system are shown. a—Family‐wise error corrected for multiple comparisons over the whole brain (*p* < .05). b—Small volume correction over the cerebellum (*p* < .05). c—Small volume correction over the brain stem (*p* < .05).

Abbreviations: BA, Brodmann areas; MCC, multiple comparison correction.

Besides, the observation of dynamic facial expressions versus dynamic mosaics activated the bilateral posterior neocortical regions (e.g., the middle temporal gyrus; Table 1). The execution of facial actions versus rest contrast induced neocortical activity in the left motor and premotor regions (Table 2).

### 3.2 Regional brain activity common to observation/execution tasks

**FIGURE 2:**
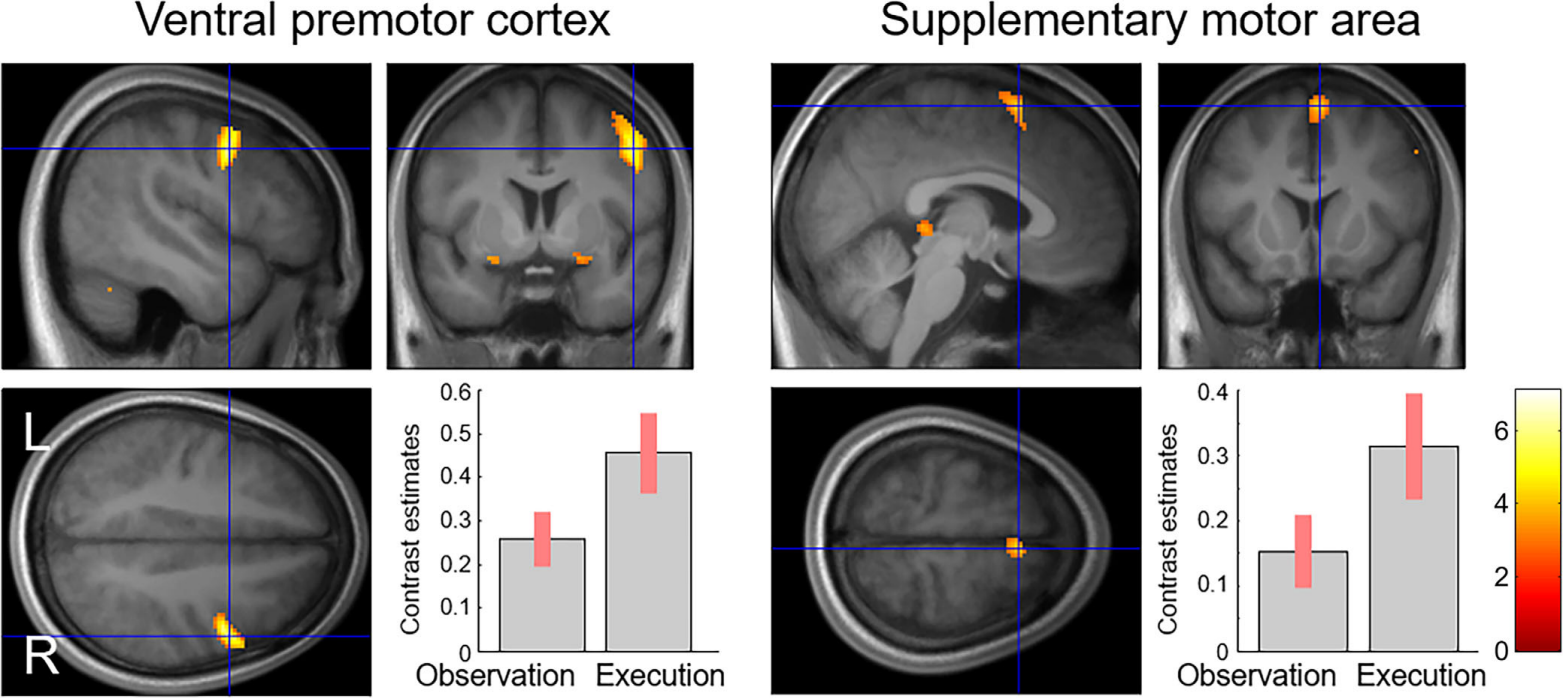
Statistical parametric maps and mean (± SE) effect size indicating shared activity in the neocortex between the observation (dynamic faces vs. dynamic mosaics) and execution tasks (facial actions vs. rest). The area is superposed on mean normalized structural images of the participants in this study.

**FIGURE 3:**
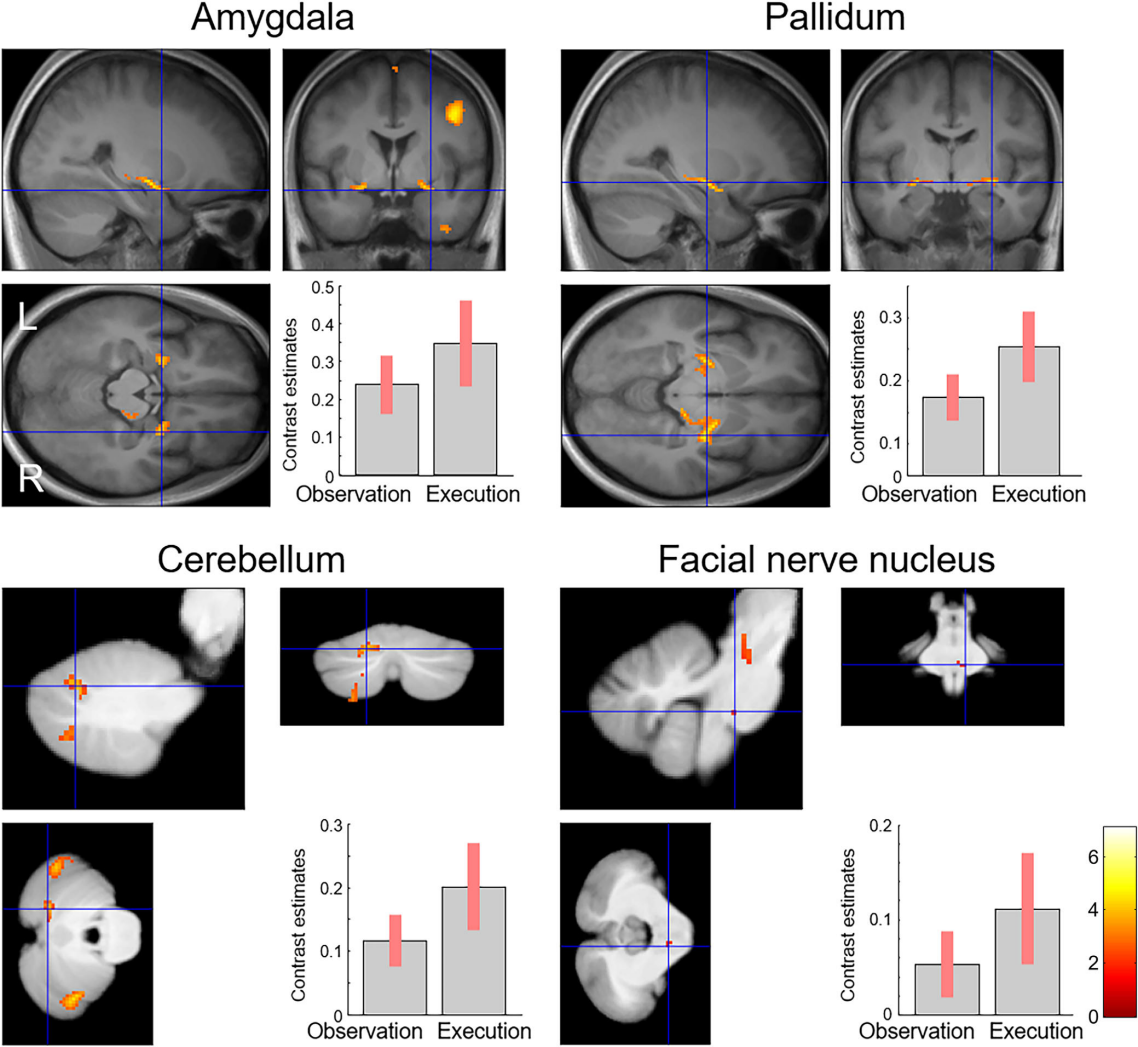
Statistical parametric maps and mean (± SE) effect size indicating shared activity in the limbic, cerebellar, and brainstem regions between the observation (dynamic faces versus dynamic mosaics) and execution tasks (facial actions versus rest). The area is superposed on mean normalized structural images of the participants in this study.

Conjunction analysis using a global null hypothesis (Friston et al., 1999) tested for commonalities in neural activity between the observation of dynamic facial expressions versus dynamic mosaics and the execution of facial actions versus rest contrasts. The results revealed significant activation in some neocortical brain regions, including the right PMv (covering the precentral gyrus and inferior frontal gyrus) and right SMA (Table 4; Figure 2). In addition, significant activation was found in the subcortical regions, including the bilateral amygdala, right basal ganglia, bilateral cerebellum, and right facial nerve nucleus (Table 4; Figure 3).

**TABLE 4:** Brain regions that exhibited significant activation in both the observation (dynamic facial expressions vs. dynamic mosaics) and execution (facial actions vs. rest) tasks.

Side	Region	BA	Coordinates			Z‐value	Cluster	MCC
			x	y	z		Size (voxel)	
R	Precentral gyrus	6	48	4	42	8.13	345	a
R	Pallidum	‐	28	−10	−8	7.74	278	a
R	Amygdala	‐	26	0	−14	6.64		a
L	Amygdala	‐	−24	2	−14	6.47	104	a
R	Supplementary motor area	6	4	14	64	6.60	130	a
L	Supplementary motor area	6	−4	8	70	4.82		a
R	Inferior temporal gyrus	20	36	−2	−44	5.68	24	a
L	Thalamus	‐	−26	−20	−6	5.23	6	a
R	Superior temporal gyrus	48	52	−38	22	4.87	2	a
R	Superior temporal gyrus	42	56	−38	20	4.72	1	a
R	Cerebellum crus 1	‐	42	−56	−27	6.96	83	b
L	Cerebellum VI	‐	−16	−72	−27	6.52	60	b
L	Cerebellum crus 1	‐	−42	−66	−27	6.51	78	b
L	Cerebellum VIIb	‐	−26	−68	−53	6.48	101	b
L	Cerebellum VIIb	‐	−12	−76	−47	5.32	33	b
L	Vermis IX	‐	−2	−54	−37	5.30	75	b
R	Cerebellum X	‐	22	−36	−41	4.62	4	b
R	Facial nerve nucleus	‐	8	−32	−37	4.84	6	c

*Note*: The coordinates of activation foci in the MNI system are shown. a—Family‐wise error corrected for multiple comparisons over the whole brain (*p* < .05). b—Small volume correction over the cerebellum (*p* < .05). c—Small volume correction over the brain stem (*p* < .05).

Abbreviations: BA, Brodmann areas; MCC, multiple comparison correction.

### 3.3 Functional brain network common to observation/execution tasks

**FIGURE 4:**
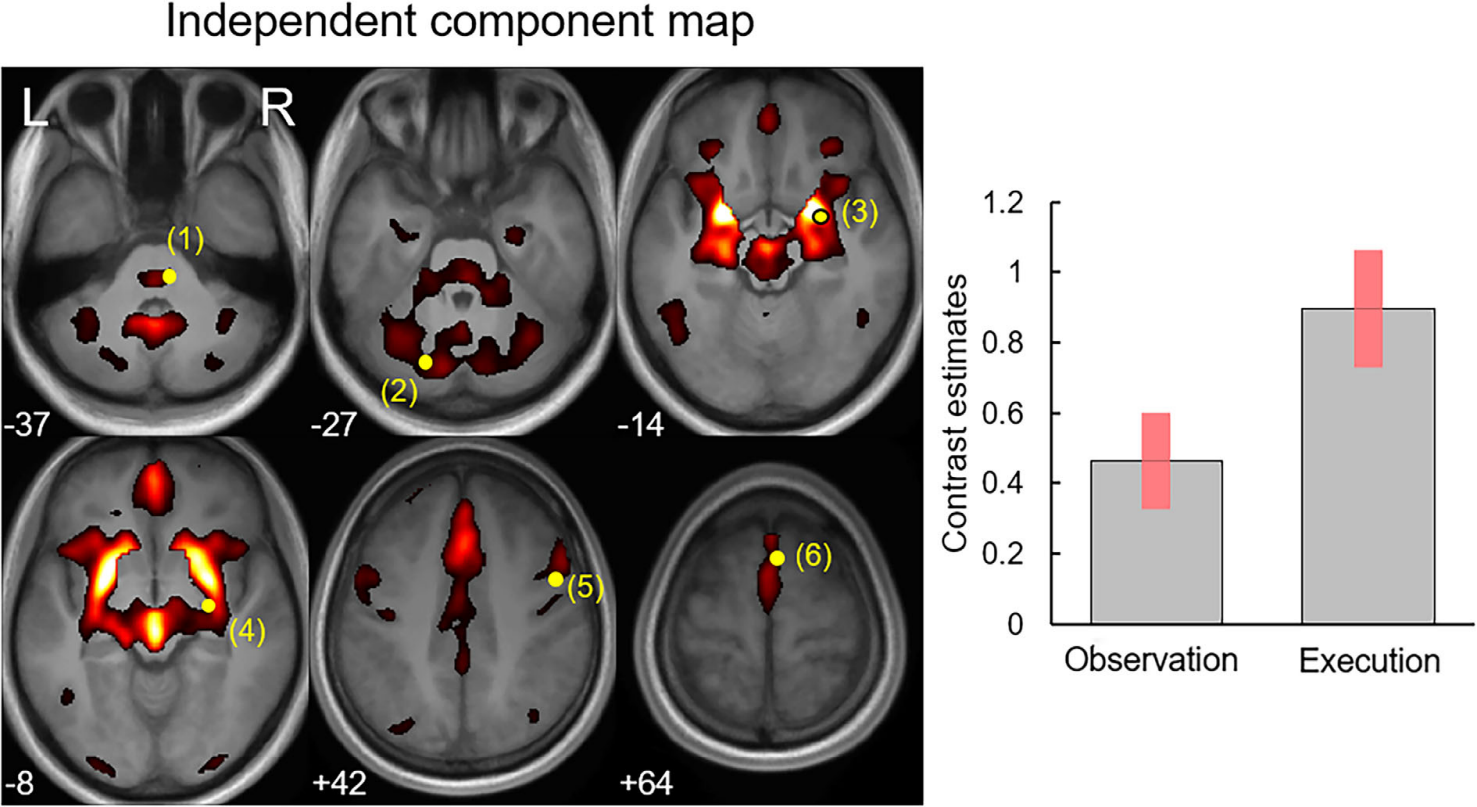
Group independent component maps (component #7) and mean (± SE) effect size of the regression analysis on this component time course indicating significant network activity in both the observation (dynamic faces vs. dynamic mosaics) and execution (facial actions vs. rest) tasks. The component network is superposed on mean normalized structural images of the study participants. Yellow circles represent activation foci detected in the conjunction analysis, including the facial nerve nucleus (1), cerebellum (2), amygdala (3), pallidum (4), ventral premotor cortex (5), and supplementary motor area (6).

Group ICA extracted independent components for the functional brain network in association with the tasks. The task‐related regression and contrast analyses for the time course of independent components revealed that only one component (component #7) was significant for both the dynamic facial expressions versus dynamic mosaics and the execution of facial actions versus rest contrasts (Figure 4). That component included widespread neocortical, limbic, cerebellar, and brainstem regions. Comparison of the spatial map of the component and activation patterns revealed by conjunction analysis using a global null hypothesis showed that the component included activation foci in the right PMv, right SMA, bilateral amygdala, right basal ganglia, bilateral cerebellum, and right facial nerve nucleus.

## 4 DISCUSSION

The results of the present study showed that the neocortical regions, specifically the PMv and SMA in the right hemisphere, were jointly activated during the observation of dynamic facial expressions versus dynamic mosaics, and during the execution of facial actions related to emotional expressions versus rest. The activation of the right PMv is in line with the previous finding that this region was consistently active during both the observation/execution of facial expressions (Anders et al., 2012; Hennenlotter et al., 2005; Kircher et al., 2013; Krautheim et al., 2020). Activation of the region around the SMA was also reported in one of these studies (Kircher et al., 2013). The activation of these neocortical regions is also consistent with results of studies testing the observation/execution matching system using nonfacial‐expression stimuli. For example, a previous functional neuroimaging study (Gazzola & Keysers, 2009) and single‐unit recording study in humans (Mukamel et al., 2010) analyzing the observation/execution of hand movements found SMA activity in both cases. Taken together with these previous data, our results suggest that the right PMv and SMA constitute the action observation/execution matching system for processing emotional facial expressions.

In addition, our results demonstrated activity common to both the observation/execution of facial expressions in several subcortical regions, including the bilateral amygdala and left cerebellum. Activation of these regions is consistent with the results of previous studies investigating the observation/execution of facial expressions (Kircher et al., 2013; Krautheim et al., 2020). These studies reported activation of the amygdala in both of these tasks. The activation of the amygdala and cerebellum is also in agreement with another study investigating the observation/execution of facial expressions (Hennenlotter et al., 2005), although overlap between the tasks was not detected. Shared neural activity between the observation/execution of facial expressions was also found in the right basal ganglion. The activation of this region has also been reported in previous studies that have tested the action observation/execution matching system using facial expressions (Anders et al., 2012) and hand gestures (Errante & Fogassi, 2020). These data suggest that the human observation/execution matching system for processing emotional facial expressions extends into these subcortical regions.

More important, our study revealed activation of the right facial nerve nucleus during both the observation/execution of facial expressions. Activation of this region during the execution of facial actions is consistent with the findings of a previous neuroimaging study (Komisaruk et al., 2002). The results are also compatible with ample behavioral evidence that the observation of dynamic facial expressions induces congruent facial muscle activity (e.g., Rymarczyk et al., 2011; Sato et al., 2008; Sato & Yoshikawa, 2007a; Weyers et al., 2006), because the facial nerve connects brain centers to facial muscles (Cattaneo & Pavesi, 2014). However, to the best of our knowledge, no neuroimaging studies have reported activation of this region during the observation of facial expressions. This study provides the first evidence that the facial nerve nucleus in the brainstem exhibits the mirror neuron property.

Furthermore, our group ICA results demonstrated that the neocortex, limbic system, basal ganglia, cerebellum, and brainstem constitute the functional network commonly activated during the observation/execution of facial expressions. The results are consistent with previous findings of functional or effective connectivity among these regions during the processing of facial expressions (e.g., Voon et al., 2010), although previous studies detected functional coupling only in part of the widespread network identified in the present study. In addition, none of the previous studies tested joint functional/effective connectivity during the observation/execution tasks. The construction of a functional network among these brain regions is in agreement with anatomical evidence of inter‐regional projections among these regions in animals (e.g., Kotchabhakdi & Walberg, 1977). Interestingly, visual inspection of the commonly activated independent component (Figure 4) suggested the possibility that more regions may be included in this network, such as the dorsomedial prefrontal cortex; this region has been reported to be associated with the processing of dynamic facial expressions and facial mimicry (Korb et al., 2019). Extending the findings from previous studies, our study revealed that widespread action observation/execution matching system regions constitute the functional network during the observation/execution of facial expressions.

Our data have theoretical significance, in that they suggest that the observation/execution matching network for the processing of emotional facial expressions encompasses widespread brain regions, including the PMv, SMA, amygdala, cerebellum, and facial nerve nucleus. As mirror neurons were first found in the PMv (Grafton et al., 1996; Rizzolatti et al., 1996), some previous theories implicated the action observation/execution matching system in the neocortex in the processing of facial emotional expressions (e.g., Williams et al., 2020). However, the present results suggest that mirror neurons are widely distributed in the brain, even in low‐level regions such as the facial nerve nucleus. This may explain why infants, whose neocortical regions might not be fully developed, show facial mimicry in response to dynamic facial expressions (Hashiya et al., 2019; Isomura & Nakano, 2016; Kaiser et al., 2017; Soussignan, 2018). One method for testing this hypothesis may be to investigate the facial mimicry ability of anencephalic patients. It has been reported that anencephalic infants, who generally develop neither the neocortex, limbic regions, nor cerebellum (Radford et al., 2019), show spontaneous facial expressions such as smiling and crying (Dickman et al., 2016; Melnick et al., 1987; Shewmon et al., 1999); this suggests that their facial nerve nucleus can catalyze the production of facial expressions. If these patients are capable of facial mimicry, the facial nerve nucleus would be the primary candidate responsible for matching between the observation/execution of facial expressions. Further research is needed to gain a deeper understanding of the functional significance of the neocortical, limbic, cerebellum, and brainstem action observation/execution matching system.

Several limitations of this study should be acknowledged. First, although we observed action observation/execution matching system activity during the observation task, it may not have been optimal. As described above, several previous psychological studies found that human observers automatically demonstrate congruent facial muscular activity while observing others' facial expressions (e.g., Dimberg, 1982). Therefore, our participants may have automatically activated their facial muscles during observation, and action observation/execution matching regional activity may reflect execution‐related activity. This issue is important because we did not measure participants' facial muscle activity, unlike some previous fMRI studies (e.g., Korb et al., 2019; Likowski et al., 2012; Rymarczyk et al., 2018). However, automatic motor synchronization can occur during the observation of not only facial expressions, but also bodily actions such as arm movements (e.g., Berger & Hadley, 1975; for a review, see Mogan et al., 2017). Automatic facial mimicry in response to conspecifics' facial expressions has been reported to occur even in nonhuman primates, including apes (Davila‐Ross et al., 2008; Palagi et al., 2019) and monkeys (Mancini et al., 2013). These data suggest that previous neurophysiological and neuroimaging findings of primates' action observation/execution matching system activity during the observation of others' actions may at least partially reflect automatic motor synchronization. Further studies are needed to evaluate the contribution of motor synchronization to action observation/execution matching system activity during the observation of others' actions.

Second, our findings could not identify the psychological functions of the action observation/execution matching system other than activation during the observation/execution of facial expressions. This issue is particularly relevant to the amygdala, which is not generally considered a part of the motor system (Holstege, 1998). Previous theoretical studies have provided different perspectives on action observation/execution matching system‐like activity in limbic regions. For example, Gallese and colleagues proposed that the embodied simulation is general and accomplishes nonmotor processing, including of emotional experiential understanding (Gallese, 2005; Gallese et al., 2004; Gallese & Caruana, 2016). By contrast, Wood et al. (2016) suggested that the observation of others' emotional expressions initially activates the sensorimotor system, which in turn facilitates emotion recognition. These accounts may not be mutually exclusive, and several other functions of the action observation/execution matching system have been proposed (Kilner & Lemon, 2013). Adding tasks to the observation/execution tasks may enhance or suppress the activity of certain observation/execution matching regions, and thus may allow inference of their functional properties. For example, a previous fMRI study reported that the participants' attention toward the emotional content, rather than the color, of dynamic bodily expressions of emotion enhanced amygdala activity (Pichon et al., 2012). Another study demonstrated that intentional imitation, compared with passive observation, of negative facial expressions facilitated amygdala activity in response to facial expressions (Hennenlotter et al., 2009); participants who had received a botulinum toxin injection into their corrugator supercilii muscles showed reduced amygdala activity during the imitation of negative facial expressions compared with participants who did not receive the injection. These data suggest that amygdala action observation/execution matching function may be a reflection of emotional simulation or the result of sensorimotor simulation. Future studies should engage participants in active tasks related to dynamic facial expressions to elucidate the functions of the action observation/execution matching system.

Finally, we detected only a small cluster (i.e., six voxels) of activation in the right facial nerve nucleus during both the observation/execution tasks. This may have been due to the difficulty in fMRI investigations of brainstem activity, including the small size of the structures of interest and high physiological noise (Beissner, 2015; Beissner et al., 2014; Brooks et al., 2013). We believe that the ICA results, which were obtained using a different approach but demonstrated the involvement of the same region, corroborate the findings of regional activation analysis. However, further studies aiming to measure facial nerve nucleus activity using sophisticated methods, such as the use of brainstem‐optimized coils (Cohen‐Adad et al., 2011), are warranted to explore the functions of this region in facial expression processing.

In conclusion, our results showed that, in addition to the neocortical regions including the right PMv and SMA, the bilateral amygdala, right basal ganglia, bilateral cerebellum, and right facial nerve nucleus were activated during both the observation/execution expression tasks. Our results further demonstrated that these regions constitute the functional network commonly activated during the observation/execution of facial expressions. These data suggest that the widespread action observation/execution matching network, involving the neocortex, limbic system, basal ganglia, cerebellum, and brainstem, is involved in the processing of facial expressions.

## CONFLICT OF INTEREST STATEMENT

The authors declare no competing financial or other interests.

## Data Availability

The data that support the findings of this study are available on request from the corresponding author. The data are not publicly available due to ethical restrictions.
